# Change of vitamin D status and all-cause mortality among Chinese older adults: a population-based cohort study

**DOI:** 10.1186/s12877-022-02956-1

**Published:** 2022-03-24

**Authors:** Jing Zeng, Ting Li, Banruo Sun, Xinyu Miao, Lin Wang, Li-chao Ma, Nan Li, Yanping Gong, Yao He, Chunlin Li, Miao Liu

**Affiliations:** 1 Department of Endocrinology, the Second Medical Centre & National Clinical Research Centre for Geriatric Disease, Chinese PLA General Hospital, 28 Fuxing Road, Beijing, 100853 China; 2 Institute of Geriatrics, Beijing Key Laboratory of Aging and Geriatrics, State Key Laboratory of Kidney Diseases, National Clinical Research Center for Geriatrics Diseases, Second Medical Center of Chinese PLA General Hospital, 28 Fuxing Road, Beijing, 100853 China; 3 Graduate School of Chinese, PLA General Hospital, 28 Fuxing Road, Beijing, 100853 China

**Keywords:** Vitamin D deficiency, Mortality, Cohort study, Aged, Change

## Abstract

**Background:**

The association of vitamin D with all-cause mortality remains controversial and longitudinal evidence exploring the potential effects of change in vitamin D status is limited in the oldest old (aged ≥ 80 years old). We aimed to study the relationship between vitamin D change and all-cause mortality among older Chinese adults including the oldest old.

**Methods:**

The data of Chinese Longitudinal and Health Longevity Study in 2012 and 2014 wave was used for baseline data. Mortality was assessed in the subsequent 2018 survey waves. Cox proportional hazard regression models were used to calculate hazard ratios (HRs) and 95% confidence interval (CI) of all-cause mortality related to vitamin D change, including maintaining deficiency or no deficiency, deficiency to no deficiency, and no deficiency to deficiency, using below 50 nmol/L as definition of deficiency.

**Results:**

The mean age of the total 1362 participants was 84.4 ± 12.1(60–113) years. The prevalence of vitamin D deficiency was 67.5% and 68.4% in 2012 and 2014 wave respectively, and significantly differed by sex and age at baseline. Cox regression showed that participants with deficiency to no deficiency and maintaining no deficiency of vitamin D status had decreased HR for all-cause mortality, compared to the maintaining deficiency group. The HRs for mortality were 0.70(95%CI: 0.50–0.96, *p* = 0.028) and 0.47(95%CI: 0.33–0.68, *p* < 0.001) respectively in the adjusted model. Also, females and the oldest old had a greatest reduction in mortality risk. And no significant difference in mortality in the no deficiency to deficiency group.

**Conclusions:**

Not only maintaining no deficiency, but also the change from deficiency to no deficiency of vitamin D status were associated with lower risk of all-cause mortality, especially in the female and oldest-old participants initially with low vitamin D level.

**Supplementary Information:**

The online version contains supplementary material available at 10.1186/s12877-022-02956-1.

## Introduction

Vitamin D has many skeletal and extra-skeletal benefits for the human body, and has been of much interest in the era of the Covid [1–3]. However, vitamin D deficiency is quite common in older adults, and the prevalence reaches as high as 70% (leave out 2–70%) in Southeast Asians [4], varying by sex and age [5]. The situation may be exacerbated in China where most people are accustomed to using sun protection, such as umbrellas, sunglasses and hats and have low awareness of vitamin D supplementation [6, 7].

Previous meta-analyses based on real-world cohort studies of adults have demonstrated a beneficial and inverse association between vitamin D and all-cause mortality [8, 9], although the shape (linear [10], non-linear [11], or U-shaped [12]) of this association is still controversial. However, null effects were found in Mendelian randomization studies, which evaluated the genetically lowered serum 25OHD concentrations on various health outcomes [13]. Moreover, RCT studies of vitamin D supplementation have also showed only weak or no benefits [14]. One plausible reason is the difference in baseline vitamin D status, treatment regimens, dosing intervals, and limited follow-up time, and some studies had individuals with higher baseline vitamin D status. That is to say, the existing evidence is inconclusive as regards any effect of vitamin D on mortality, particularly in oldest old who are aged ≥ 80 years old and under-represented in studies.

In addition, the vitamin D status, represented by circulating 25-hydroxyvitamin D (25(OH)D) concentration, is not fixed over time, along with the change of nutritional status, sunlight exposure and comorbidities [15, 16]. But previous longitudinal studies have more focused on baseline vitamin D level, and few studies have addressed the association of serum 25(OH)D change with mortality. Hence, this study is aimed to prospectively examine the sex and age-specific association between the change of vitamin D status and all-cause mortality among a group of community older adults and especially with regard to the oldest old, using data from the Chinese Longitudinal Healthy Longevity Survey (CLHLS).

## Methods

### Ethical approval

The study was approved by the Ethics Committee of Duke University and Peking University (No. IRB00001052-13,074). Informed consent was signed by each participant or their legal representatives prior to data collection.

### Study population

**Fig. 1:**
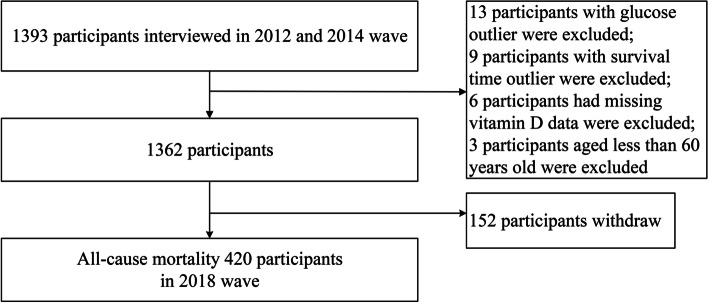
Flow chart of inclusion of participants

All data of our study was from the Chinese Longitudinal Healthy Longevity Survey (CLHLS), a nationwide prospective cohort study based on representative community population of each age group to explore the determinants for healthy aging of China. The detail methods are presented in previous studies [17, 18]. We included participants who had completed questionnaire and blood biochemical tests in both the 6th wave (2012) and the 7th (2014) the survey, and followed in the 8th (2018) wave of CLHLS in eight longevity areas. A total of 1,393 participants were initially in the 2012 and 2014 baseline, and then participants who had outliers of glucose (*n* = 13) and survival time (*n* = 9), incomplete vitamin D data (*n* = 6) and aged less than 60 years (*n* = 3) were excluded. Thus, in the final analysis we included 1,362 participants (661 male and 701 female aged 60 to 113 years old), and 152 of them were lost to follow-up in 2018 wave. The flow chart was showed in Fig. 1.

### Assessment of vitamin D and covariates

Fasting blood samples of each participant were collected by trained nurses. All collected blood samples were centrifuged and transported to Beijing for uniform testing.

Serum 25(OH)D levels were measured by using the enzyme-linked immunosorbent assay (Immunodiagnostic Systems Limited, Bolton, UK). The inter- and intra-assay coefficients of variation were < 10% and < 8%, respectively [19]. The definition of vitamin D deficiency is varied and still not universal consensus [20]. According to the definition of Endocrine Society Guidelines [21], the cutoff point 50 nmol/L was used and classified 25 (OH) D level into two categories: deficiency, less than 50 nmol/ L; 50 nmol/L or above, no deficiency. Change of vitamin D status was therefore classified into four types: (1) maintaining deficiency, the levels were < 50 nmol/L in 2012 and 2014 wave; (2) no deficiency to deficiency, the level was ≥ 50 nmol/L in 2012 and changed to < 50 nmol/L in 2014; (3) deficiency to no deficiency, the level was < 50 nmol/L in 2012 and changed to ≥ 50 nmol/L in 2014; and (4) maintaining no deficiency, the levels were ≥ 50 nmol/L in 2012 and 2014 wave.

A standardized questionnaire including demographic and sociological characteristics, lifestyle and disease history was conducted by trained interviewers. Our study included the following covariates in 2014 baseline: sex, age, married and living with spouse, residence (rural vs. urban), education (illiteracy vs. 1–6 years and vs. ≥ 7 year of schooling), exercise (never vs. ever), smoking (never vs. ever), drinking (never vs. ever), poor self-rated health (by answering “self-reported health” with bad or very bad), history of cardiac or cerebrovascular disease (by answering “self-reported heart disease, and stroke/cerebrovascular disease” with yes vs. no), score of Mini-Mental State Examination (MMSE), waist circumference (WC), systolic and diastolic blood pressure (SBP and DBP) and serum hemoglobin (HGB), albumin (Alb), fasting blood-glucose (FBG), creatinine, uric acid and blood lipid level.

### Outcome measures

Mortality was ascertained during the subsequent 2018 wave of CLHLS. The date of death was collected from officially issued death certificates or the next of kin. The follow-up time was measured from the interview date of 2014 wave to the date of death. Survival is defined as not being traced to death and surveyed in the 2018 wave. Their follow-up time was measured as the interval of interview date between 2014 and 2018 wave. For participants who were lost, the follow-up time was calculated as the half of time between the interview date of 2014 wave and the date when loss of follow-up was found.

### Statistical analysis

Statistical analysis was performed using SPSS 19.0 and Stata 16.0. Given the non-normality of variables, median (interquartile range, IQR) was used for continuous variables and N (%) was used for categorical variables. Kruskal–Wallis test and Chi-square test were used to compare univariate difference among four types of vitamin D change. Multivariable Cox proportional risk model was then performed to assess the association of vitamin D change and all-cause mortality, after testing the proportional risk model assumptions. The hazard ratio (HR) and 95% confidence was calculated adjusted for statistically and clinically significant variables. A predefined subgroup stratified by sex and age (aged 60–79 years,80–99 years and ≥ 100 years) was conducted. Considering the inclusion of some oldest-old participants, we further conducted sensitivity analyses to test the robustness of the primary results: (1) excluding participants died within half a year of follow-up; and (2) excluding participants with poor self-rated health; (3) excluding participants with cardiac cerebrovascular disease. A two-sided *P* value < 0.05 was considered statistically significant.

## Results

A total of 1362 participants were included in our analysis. The median level of 25(OH)D was 40.69(24.88) nmol/L and 39.30(26.73) nmol/L in 2012 and 2014 wave, respectively. The prevalence of vitamin D deficiency was similar in both waves (67.5% vs. 68.4%) and significantly differed by sex and age (*p* < 0.001), and more than 70 percent of female and participants aged 80 years old or above had vitamin D deficiency in each wave (Table S1, see additional file). Similar results can be seen in the vitamin D change, and female and older participants were more likely to maintain deficiency (Table 1). The proportion maintaining deficiency, no deficiency to deficiency, deficiency to no deficiency and maintaining no deficiency was 704(51.7%), 227(16.7%), 215(15.8%) and 216(15.9%), respectively.

**Table 1:** General characteristics of the 1,362 participants with different vitamin D changes

	**Vitamin D change**				
	**Deficiency** **(*n* = 704)**	**No deficiency to deficiency** **(*n* = 227)**	**Deficiency to no deficiency** **(*n* = 215)**	**No deficiency** **(*n* = 216)**	***P*-value**
**Median (IQR)**					
**Age, years**	87.5(78.0–97.0)	79.0(72.0–88.5)	79.0(71.0–88.0)	81.5(72.0–89.0)	< 0.001
**SBP, mmHg**	141(128–160)	140(126–160)	140(125–150)	140(128–150)	0.067
**DBP, mmHg**	80(71–89)	80(71–90)	80(77–90)	80(71–88)	0.180
**WC, cm**	80.0(74.0–88.0)	83.0(76.0–90.0)	81.0(75.5–89.0)	79.0(73.0–84.0)	< 0.001
**HGB, g/L**	125.00(113.00–136.00)	130.00(119.00–140.00)	140.00(127.50–152.00)	129.00(114.00–142.00)	< 0.001
**Alb, g/L**	42.60(39.80–44.70)	43.20(41.00–45.25)	43.80(40.85–45.50)	43.80(41.80–45.62)	< 0.001
**FBG, mmol/L**	5.01(4.45–5.75)	4.94(4.39–5.82)	5.27(4.67–5.87)	4.89(4.01–5.50)	< 0.001
**Creatinine, mmol/L**	75.45(65.17–92.12)	79.40(68.65–92.30)	74.80(66.15–86.80)	80.30(69.17–94.65)	0.068
**Uric acid, μmol/L**	272.00(224.00–327.00)	296.00(253.50–344.50)	272.00(235.00–320.00)	314.00(264.00–380.50)	< 0.001
**TC, mmol/L**	4.78(4.17–5.51)	4.85(4.25–5.49)	4.48(3.96–5.09)	4.62(4.06–5.12)	< 0.001
**TG, mmol/L**	1.06(0.80–1.48)	1.07(0.80–1.50)	1.07(0.83–1.58)	0.95(0.75–1.37)	0.181
**HDL-c, mmol/L**	1.36(1.11–1.65)	1.42(1.17–1.65)	1.25(1.03–1.55)	1.42(1.18–1.66)	< 0.001
**25(OH)D 2012, nmol/L**	32.60(25.66–40.41)	58.90(53.91–66.67)	34.00(27.23–42.27)	63.92(56.38–75.07)	< 0.001
**25(OH)D 2014, nmol/L**	30.45(21.90–38.23)	36.80(31.35–42.80)	61.20(55.15–72.10)	65.35(57.50–75.93)	< 0.001
**MMSE**	24.00(16.00–29.00)	28.00(24.00–29.00)	27.00(22.00–29.00)	26.50(23.00–29.00)	< 0.001
**N (%)**					
**Age, years**					< 0.001
60–79	202(28.7)	115(50.7)	109(50.7)	97(44.9)	
80–99	358(50.9)	87(38.3)	80(37.2)	95(44.0)	
≥ 100	144(20.5)	25(11.0)	26(12.1)	24(11.1)	
**Sex**					< 0.001
Male	244(34.7)	133(58.6)	139(64.7)	145(67.1)	
Female	460(65.3)	94(41.4)	76(35.3)	71(32.9)	
**Currently married and living with spouse**	231(32.8)	127(55.9)	116(54.0)	100(46.3)	< 0.001
**Residence**					< 0.001
Rural	546(77.6)	171(75.3)	199(92.6)	199(92.1)	
Urban	158(22.4)	56(24.7)	16(7.4)	17(7.9)	
**Education**					< 0.001
Illiteracy	461(65.9)	99(44.4)	125(58.1)	111(52.1)	
≤ 6yrs	181(25.9)	89(39.9)	62(28.8)	72(33.8)	
≥ 7yrs	58(8.3)	35(15.7)	28(13.0)	30(14.1)	
**Current/past drinking**	119(16.9)	65(28.6)	42(19.5)	56(25.9)	< 0.001
**Current/past smoking**	130(18.5)	72(31.7)	46(21.4)	66(30.6)	< 0.001
**Current/past exercise**	109(15.5)	38(16.7)	43(20.0)	35(16.2)	0.482
**Poor self-rated health**	82(11.6)	29(12.8)	23(10.7)	21(9.7)	0.759
**History of cardiac or cerebrovascular disease**	116(16.5)	23(10.1)	43(20.0)	14(6.5)	< 0.001

Data were expressed as median (interquartile range, IQR) or n (%). *SBP* systolic blood pressure, *DBP* diastolic blood pressure, *WC* waist circumference, *HGB* hemoglobin, *Alb* albumin, *FBG* fasting blood-glucose, *TC* total cholesterol, *TG* total triglyceride, *HDL-c* high density lipoprotein cholesterol, *MMSE* Mini-Mental State Examination

### Demographic and symptomatic characteristics of each vitamin D change group

Baseline characteristics of the participants were presented in Table 1. The participants with maintaining deficiency of serum 25(OH)D were more likely to be older, be female, have less proportion of currently married and living with a spouse, have less education, have less smoking or alcohol usage, have a lower MMSE score, have lower HGB and Alb level. While participants with maintaining no deficiency of serum 25(OH)D were more likely to be male, be in rural, have higher uric acid level, have higher HDL-c level, and have lower history of cardiac cerebrovascular disease. No difference was found in blood pressure, creatinine, TG and poor self-rated health (*p* > 0.05).

### All-cause mortality of each vitamin D change group

There was a total of 420 mortality cases during the 3675.8 person-years. The total all-cause mortality was 30.84% and the corresponding incidence density was 11.43 per 100 person-years. As we can see from Table 2, participants with maintaining deficiency of vitamin D were 2.33-fold as likely to have the all-cause mortality as those with maintaining no deficiency (39.99% Vs.17.13%, *p* < 0.001). Specially, participants who were with vitamin D deficiency at follow-up (including deficiency and no deficiency to deficiency) had a relatively higher all-cause mortality than those who were with no deficiency.

**Table 2:** All-cause mortality according to Vitamin D change

	**Cases and mortality cases**	**Mortality (%)**	**Total person-years**	**Mortality density (per 100 person-years)**
Total	1362(420)	30.84	3675.80	11.43
Vitamin D change				
Deficiency	704(281)	39.99*	1778.96	15.80*
No deficiency to deficiency	227(57)	25.11	661.33	8.62
Deficiency to no deficiency	215(45)	20.93	598.88	7.51
No deficiency	216(37)	17.13	636.63	5.81

^*^Comparison between groups *P* < 0.001

### HRs of vitamin D change for all-cause mortality

Table 3 showed the HRs and 95% CI of vitamin D change for all-cause mortality. After adjusted for age, sex, SBP, WC, smoking, drinking, education, married status, exercise, residence, poor self-rated health, MMSE, history of cardiac-cerebral vascular disease, HGB, FBG, creatinine, uric acid and TC in the model, the HRs of no deficiency to deficiency, deficiency to no deficiency, and maintaining no deficiency were 0.78(95%CI: 0.58–1.05), 0.70(95%CI: 0.50–0.96) and 0.47(95%CI: 0.33–0.68), respectively compared to maintaining deficiency group.

**Table 3:** Hazard ratios for the association between vitamin D change and all-cause mortality (*n* = 1,362)

All-cause mortality	Vitamin D change						
	**Deficiency**	**No deficiency to deficiency**	***p* value**	**Deficiency to no deficiency**	***p* value**	**No deficiency**	***p* value**
Number/deaths	704/281	227/57		215/45		216/37	
Crude Model	1(Ref.)	0.54(0.41–0.72)	< 0.001	0.47(0.34–0.64)	< 0.001	0.36(0.26–0.51)	< 0.001
Model 1	1(Ref.)	0.71(0.53–0.95)	0.022	0.63(0.46–0.87)	0.005	0.45(0.32–0.64)	< 0.001
Model 2	1(Ref.)	0.77(0.68–1.03)	0.076	0.64(0.46–0.88)	0.006	0.47(0.33–0.67)	< 0.001
Model 3	1(Ref.)	0.78(0.58–1.05)	0.097	0.70(0.50–0.96)	0.028	0.47(0.33–0.68)	< 0.001

Model 1. adjusted for age and sex

Model 2. adjusted for model1 + SBP, WC, smoking, drinking, education, currently married and living with spouse, exercise, residence, poor self-rated health, MMSE and history of cardiac cerebrovascular disease

Model 3. adjusted for Model2 + HGB, FBG, creatinine, uric acid and TC

**Fig. 2:**
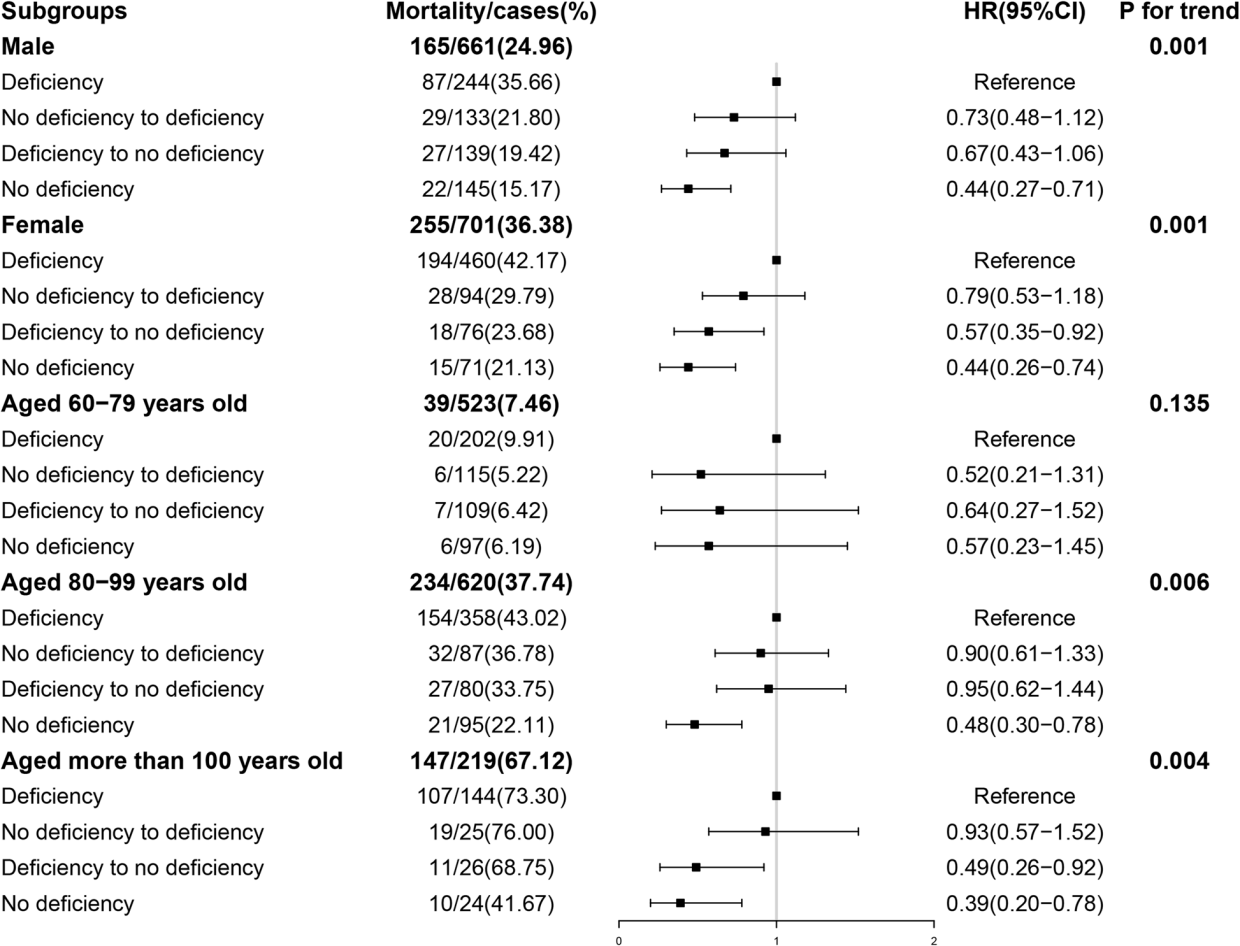
Hazard ratios for the sex and age-specific association between vitamin D change and all-cause mortality (*n* = 1,362)

Compared with different sex, as can be seen from Fig. 2 and Table S2(see additional file), the association was robust and remarkable among female. The HRs of deficiency to no deficiency and maintaining no deficiency were 0.57(95%CI: 0.35–0.92) and 0.44(95%CI: 0.26–0.74), respectively. In male, the significant association between vitamin D change and all-cause mortality only observed in maintaining no deficiency group, similarly as the result of female.

When further compared with different ages (Fig. 2, Table S3 see additional file), the estimated benefit for mortality was stronger in participants aged ≥ 80 years than participants aged 60–80 years. The adjusted HRs of deficiency to no deficiency and maintaining no deficiency were 0.49(95%CI:0.26–0.92) and 0.39(95%CI: 0.20–0.78) in the oldest-old group. While the result of maintaining no deficiency group in participants aged 80–99 years was corresponding to those of total.

And no significance was observed in the no deficiency to deficiency group, regardless of sex and age. All sensitivity analyses showed similar results (Table S4, see additional file).

## Discussion

In this prospective longitudinal study based on 1362 Chinese older adults aged 60 years or older, a relatively high prevalence of vitamin D deficiency was found. And we observed the change from deficiency to no deficiency or maintaining no deficiency of vitamin D significantly reduced the all-cause mortality, especially in female and oldest-old participants. The association was independent of traditional risk factors, including demographic characteristics, lifestyle factors, cognitive, metabolic and health status. Sensitivity analyses supported the robustness of the observed associations.

Vitamin D deficiency is highly prevalent worldwide, even in countries with abundant sun exposure [15]. The reported prevalence was 2–70% in Southeast Asians [4], and was much higher in the Chinese elderly, ranging from 69.2–94.3% [22]. In this study, the total prevalence of vitamin D deficiency was corresponding to previous reports and also varied by age and sex, with females and the oldest old being more prone to deficiency. Differences in diet, sunscreen use and supplement use by age and sex, skin pigmentation and reduced skin responsiveness to ultraviolet with age might partly explain this variation [15].

At present, many prospective studies have focused on baseline vitamin D and mortality, potentially based on the premise that the baseline change during follow-up is small, or the effect of those change is not significant, which is suggested in a previous cohort study about the time-dependent relationship between vitamin D and all-cause mortality [23]. There is relatively consistent evidence that an inverse association between vitamin D and all-cause mortality [8], although null correlation has reported in some studies [24]. In this study, we considered the changes of vitamin D status, and the subgroups of the maintaining deficiency and no deficiency group accorded with the assumption of previous cohort studies. Compared with maintaining vitamin D deficiency group, maintaining no deficiency of vitamin D was associated with 53% lower risk of all-cause mortality, in line with the previous studies reported benefits range from 17 to 57% [9, 25]. Moreover, this association was more noticeable in participants aged ≥ 80 years and female, and similar results have shown in previous study from the Newcastle 85 + female study [26]. Also, previous studies on the association in males were inconsistent [27, 28]. In summary, the present study strongly supports a protective effect of vitamin D on all-cause mortality. It also suggests that optimizing vitamin D status (i.e., going from deficiency to replete status) may increase survival even over a relatively short follow up period.

As we know, observational studies were unable to assess causality. However, previous reviews based on vitamin D supplementation trials have showed a week benefit on mortality [29]. Two recent systematic reviews of such trials confirmed that vitamin D supplementation has no effect on all-cause mortality, in discordance with the prevailing results of observational studies [30, 31]. However, heterogeneity exists among different studies, and mortality is only as a secondary endpoint in some trials. Additionally, there is lack of studies in oldest old. Similar controversy emerges from mendelian randomization studies [32–34]. Our prospective study focused on the change of vitamin D status and provided evidence that elevating vitamin D to no deficiency status and/or maintaining no deficiency status was beneficial in reducing by between 30% all-cause mortality risk, and greater benefit in the oldest old and females may be due in part having lower baseline vitamin D levels. Moreover, the descent degree of correct to no deficiency was lower than those of maintaining no deficiency group, implying that the earlier it is corrected, the greater benefit. Summarily, current studies have not provided enough evidence to support causality in the relationship between vitamin D and mortality which remains inconclusive. More clinical trials (such as the ongoing VIDAL study [35]) are required to explore this, with our study suggesting that older adults (especially the oldest old, with vitamin D levels < 50 nmol/L) are the most suitable to target for supplementation where benefit may be greatest.

The protective effect of vitamin D on mortality may be attributed to a set of biological mechanisms of 1,25(OH)2 D-VDR complex, an activated form of vitamin D bound to receptors, including effects on anti-oxidative activity, cell proliferation, differentiation and apoptosis, mitochondrial function and immunoregulation [36, 37]. A recent study further revealed that vitamin D was an unique marker to healthy aging and can neither be fully explained by aging of the epigenome, loss of telomeres, or anti-oxidative effects of vitamin D metabolites [1].

This study has several strengths. CLHLS is a representative prospective study that primarily included older adults and the oldest old, and had multiple long-term follow-ups. Thus, we have the chance to explore the effect of vitamin D change on mortality, different from most prevailing studies where only baseline vitamin D status was assessed. However, there were several limitations in this study. First, even considering vitamin D changes, the casual relationship could not be confirmed in an observational design. Second, blood tests were only done on a subset of the whole sample. The wide confidence interval of HRs among younger old might diminish, if every participant got tested. Third, as a secondary data analysis, we can only analyze the association with all-cause mortality due to the lack of detail on specific causes of death in the database. Fourth, although multiple variables were adjusted, our study database also didn’t have detailed information on prescribed or over the counter vitamin D supplements, frequency and capacity of sun exposure and dairy products which might have impact on serum 25(OH)D levels. Of note, absence of more details on chronic diseases could also influence vitamin D status and all-cause mortality. And lifestyle changes over time may impact on disease risk and vitamin D status itself which could complicate any relationship with mortality. However, even when all the factors are taken into account, it is still difficult to explain most of the individual variation. For this reason, we looked at the change in vitamin D status as a predictor. Despite this, further changes in vitamin D levels at further follow up in our study which were not measured has the potential to alter results. And future studies need to take more account of chronic disease and lifestyle changes in older adults.

## Conclusion

This cohort study showed that maintaining no deficiency status of vitamin D was associated with lower risk of all-cause mortality risk, and the change of vitamin D status from deficiency to no deficiency can also reduce the mortality risk, especially in the female and oldest-old participants initially with low vitamin D level. Hence, proper attention should be paid to addressing vitamin D deficiency of older adults in clinical practice for the improvement of longevity and healthy aging. Future clinical trials targeted the sex and age-specific association between vitamin D supplementation and mortality in the old adult are still essential.

## Supplementary Information

**Additional file 1.**

## Data Availability

All data generated or analyzed during this study are included in this published article. The database of the current study is available at https://opendata.pku.edu.cn/ with permission.

## Abbreviations

IQR: Interquartile range
SBP: Systolic blood pressure
DBP: Diastolic blood pressure
WC: Waist circumference
HGB: Hemoglobin
Alb: Albumin
FBG: Fasting blood-glucose
TC: Total cholesterol
TG: Total triglyceride
HDL-c: High density lipoprotein cholesterol
MMSE: Mini-Mental State Examination
