# Supplementary material for: Change of vitamin D status and all-cause mortality among Chinese older adults: a population-based cohort study
Source: BMC Geriatr. 2022 Mar 24;22:245. doi: 10.1186/s12877-022-02956-1 (PMC8944012; doi:10.1186/s12877-022-02956-1)
Supplement: Supplementary file 1 — Additional file 1. [file 12877_2022_2956_MOESM1_ESM.docx]

Supplement table1. Prevalence of vitamin D deficiency in 2012 and 2014 wave

|  | 2012 wave | | p value | 2014 wave | | p value |
| --- | --- | --- | --- | --- | --- | --- |
|  | Deficiency | No deficiency |  | Deficiency | No deficiency |  |
| Total | 919(67.5) | 443(32.5) |  | 931(68.4) | 431(31.6) |  |
| Male | 383 (57.9) | 278(42.1) | <0.001 | 377(57.0) | 284(43.0) | <0.001 |
| Female | 536(76.5) | 165(23.5) |  | 554(79.0) | 147(21.0) |  |
| Aged 60-79 years | 311(59.5) | 212(40.5) | <0.001 | 317(60.6) | 206(39.4) | <0.001 |
| Aged 80-99 years | 438(70.6) | 182(29.4) |  | 445(71.8) | 175(28.2) |  |
| Aged ≥100 years | 170(77.6) | 49(22.4) |  | 169(77.2) | 50(22.8) |  |

Supplement table2. Hazard ratios for the sex-specific association between vitamin D change and all-cause mortality(n=1,362).

| **All-cause mortality** | **Vitamin D change** | | | | | | |
| --- | --- | --- | --- | --- | --- | --- | --- |
|  | **Deficiency** | **No deficiency to deficiency** | **p value** | **Deficiency to no deficiency** | **p value** | **No deficiency** | **p value** |
| **Male** |  |  |  |  |  |  |  |
| Number/deaths | 244/87 | 133/29 |  | 139/27 |  | 145/22 |  |
| Crude Model | 1(Ref.) | 0.54(0.36-0.83) | 0.005 | 0.48(0.31-0.75) | 0.001 | 0.37(0.23-0.59) | <0.001 |
| Model 1 | 1(Ref.) | 0.71(0.47-1.09) | 0.117 | 0.69(0.45-1.06) | 0.093 | 0.47(0.29-0.75) | 0.002 |
| Model 2 | 1(Ref.) | 0.74(0.48-1.13) | 0.157 | 0.58(0.37-0.90) | 0.015 | 0.46(0.28-0.74) | 0.001 |
| Model 3 | 1(Ref.) | 0.73(0.48-1.12) | 0.146 | 0.67(0.43-1.06) | 0.084 | 0.44(0.27-0.71) | 0.001 |
| **Female** |  |  |  |  |  |  |  |
| Number/deaths | 460/194 | 94/28 |  | 76/18 |  | 71/15 |  |
| Crude Model | 1(Ref.) | 0.61(0.41-0.90) | 0.014 | 0.52(0.32-0.85) | 0.008 | 0.42(0.25-0.72) | 0.001 |
| Model 1 | 1(Ref.) | 0.74(0.50-1.10) | 0.133 | 0.57(0.36-0.93) | 0.025 | 0.44(0.26-0.74) | 0.002 |
| Model 2 | 1(Ref.) | 0.82(0.55-1.22) | 0.325 | 0.55(0.34-0.89) | 0.014 | 0.46(0.27-0.79) | 0.004 |
| Model 3 | 1(Ref.) | 0.79(0.53-1.18) | 0.247 | 0.57(0.35-0.92) | 0.022 | 0.44(0.26-0.74) | 0.002 |

Model1. adjusted for age

Model2. adjusted for model1+SBP, WC, smoking, drinking, education, currently married and living with spouse, exercise, residence, poor self-rated health, MMSE and history of cardiac-cerebral vascular disease

Model3. adjusted for Model2+ HGB, FBG, creatinine, uric acid and TC

Supplement table3. Hazard ratios for the age-specific association between vitamin D change and all-cause mortality(n=1,362).

| **All-cause mortality** | **Vitamin D change** | | | | | | |
| --- | --- | --- | --- | --- | --- | --- | --- |
|  | **Deficiency** | **No deficiency to deficiency** | **p value** | **Deficiency to no deficiency** | **p value** | **No deficiency** | **p value** |
| **Aged 60-79 years old** |  |  |  |  |  |  |  |
| Number/deaths | 202/20 | 115/6 |  | 109/7 |  | 97/6 |  |
| Crude Model | 1(Ref.) | 0.52(0.21-1.29) | 0.156 | 0.63(0.27-1.49) | 0.290 | 0.60(0.24-1.48) | 0.265 |
| Model 1 | 1(Ref.) | - |  | - |  | - |  |
| Model 2 | 1(Ref.) | 0.58(0.23-1.46) | 0.248 | 0.65(0.27-1.55) | 0.333 | 0.66(0.26-1.68) | 0.387 |
| Model 3 | 1(Ref.) | 0.52(0.21-1.31) | 0.164 | 0.64(0.27-1.52) | 0.310 | 0.57(0.23-1.45) | 0.239 |
| **Aged 80-99 years old** |  |  |  |  |  |  |  |
| Number/deaths | 358/154 | 87/32 |  | 80/27 |  | 95/21 |  |
| Crude Model | 1(Ref.) | 0.73(0.50-1.07) | 0.11 | 0.77(0.51-1.16) | 0.213 | 0.44(0.28-0.69) | <0.001 |
| Model 1 | 1(Ref.) | 0.69(0.47-1.01) | 0.055 | 0.74(0.49-1.12) | 0.157 | 0.41(0.26-0.65) | <0.001 |
| Model 2 | 1(Ref.) | 0.87(0.58-1.28) | 0.468 | 0.85(0.56-1.28) | 0.438 | 0.47(0.29-0.75) | 0.002 |
| Model 3 | 1(Ref.) | 0.90(0.61-1.33) | 0.606 | 0.95(0.62-1.44) | 0.794 | 0.48(0.30-0.78) | 0.003 |
| **Aged ≥100 years old** |  |  |  |  |  |  |  |
| Number/deaths | 144/107 | 25/19 |  | 26/11 |  | 24/10 |  |
| Crude Model | 1(Ref.) | 0.87(0.53-1.42) | 0.569 | 0.47(0.25-0.87) | 0.016 | 0.43(0.23-0.82) | 0.011 |
| Model 1 | 1(Ref.) | - |  | - |  | - |  |
| Model 2 | 1(Ref.) | 0.93(0.57-1.52) | 0.761 | 0.49(0.26-0.92) | 0.026 | 0.39(0.20-0.78) | 0.008 |
| Model 3 | 1(Ref.) | 0.93(0.57-1.52) | 0.761 | 0.49(0.26-0.92) | 0.026 | 0.39(0.20-0.78) | 0.008 |

Model1.adjusted for age and sex

Model2.adjusted for model1+SBP, WC, smoking, drinking, education, currently married and living with spouse, exercise, residence, poor self-rated health, MMSE and history of cardiac-cerebral vascular disease

Model3.adjusted for Model2+ HGB, FBG, creatinine, uric acid and TC

Supplement table4. Sensitivity analysis of association between vitamin D change and all-cause mortality.

|  | Model1 | | Model2 | | Model3 | |
| --- | --- | --- | --- | --- | --- | --- |
|  | HR (95%CI) | p value | HR (95%CI) | p value | HR (95%CI) | p value |
| **Excluding participants died within half a year of follow-up** |  |  |  |  |  |  |
| Deficiency | 1(Ref.) |  | 1(Ref.) |  | 1(Ref.) |  |
| No deficiency-deficiency | 0.69(0.50-0.95) | 0.021 | 0.74(0.55-1.01) | 0.056 | 0.73(0.53-1.00) | 0.050 |
| Deficiency-no deficiency | 0.59(0.41-0.84) | 0.003 | 0.63(0.45-0.89) | 0.009 | 0.65(0.45-0.93) | 0.018 |
| No deficiency | 0.46(0.32-0.67) | <0.001 | 0.45(0.31-0.65) | <0.001 | 0.46(0.31-0.67) | <0.001 |
| **Excluding participants with poor self-rated health** |  |  |  |  |  |  |
| Deficiency | 1(Ref.) |  | 1(Ref.) |  | 1(Ref.) |  |
| No deficiency-deficiency | 0.66(0.47-0.92) | 0.015 | 0.72(0.52-1.01) | 0.059 | 0.70(0.50-0.98) | 0.038 |
| Deficiency-no deficiency | 0.68(0.48-0.96) | 0.028 | 0.69(0.48-0.98) | 0.037 | 0.74(0.52-1.06) | 0.101 |
| No deficiency | 0.49(0.34-0.71) | <0.001 | 0.48(0.33-0.70) | <0.001 | 0.47(0.32-0.68) | <0.001 |
| **Excluding participants with cardiac-cerebral vascular disease** |  |  |  |  |  |  |
| Deficiency | 1(Ref.) |  | 1(Ref.) |  | 1(Ref.) |  |
| No deficiency-deficiency | 0.75(0.55-1.01) | 0.061 | 0.79(0.58-1.06) | 0.119 | 0.78(0.57-1.05) | 0.102 |
| Deficiency-no deficiency | 0.51(0.35-0.75) | 0.001 | 0.51(0.35-0.75) | 0.001 | 0.57(0.39-0.84) | 0.005 |
| No deficiency | 0.45(0.31-0.65) | <0.001 | 0.45(0.31-0.66) | 0.001 | 0.44(0.30-0.64) | <0.001 |

Model1.adjusted for age and sex

Model2.adjusted for model1+SBP, WC, smoking, drinking, education, currently married and living with spouse, exercise, residence, poor self-rated health, MMSE and history of cardiac-cerebral vascular disease

Model3.adjusted for Model2+ HGB, FBG, creatinine, uric acid and TC
